# Surgical repair of inferior sinus venosus defects: a novel approach with unsnared inferior vena cava

**DOI:** 10.1186/s13019-015-0359-x

**Published:** 2015-11-06

**Authors:** Fushun Lin, Hong Tang, Xijun Xiao

**Affiliations:** 1 Department of Cardiovascular Surgery, West China Hospital, Sichuan University, Guoxuexiang 37th, 610041 Chengdu, Sichuan P.R. China; 2 Cardiology Department, West China Hospital, Sichuan University, Chengdu, P.R. China

**Keywords:** Cardiac surgery, Inferior sinus venosus defect, Cannulation, Tourniquets

## Abstract

**Background:**

Inferior sinus venosus defects (SVD) are very rare and difficult to image from transthoracic echocardiography. Surgical errors were occasionally reported in the repair of inferior SVDs.

**Results:**

The authors have operated on 12 inferior SVD patients using bicaval cannulation with unsnared inferior vena cava (IVC) and proved successful.

**Conclusion:**

This technique guaranteed a better exposure of surgical field and facilitate identifying the anatomical relationship between lower part of the SVD and IVC orifice, thus avoiding postoperative IVC - left atrial shunt and other surgical mistakes.

## Background

Inferior Sinus venosus defect is a rare type of interatiral communication involving lower part of the atrial septum derived from the sinus venosus [1, 2]. The lower edge of the defect has no residual atrial septal tissue thus the orifice of IVC strides over the atrial septum [3–5]. Giant residual Eustachian valve in some patients may be mistaken as the lower edge of the defect, leading to misdiagnosis [3]. Preoperative diagnosis of inferior SVD remains challenging. TEE has been reported to be better than TTE in diagnosing inferior SVD [3, 5, 6]. Even so, the accurate preoperative diagnosis of inferior SVD is difficult, especially in medical centers where TEE cannot be routinely performed.

As the defects locate inferioposterior to the fossa ovalis and are difficult to depict by TTE preoperatively, surgical errors were occasionally reported if the anatomical correlation was not fully identified [3, 7]. A rare but serious complication is residual IVC- left atrial shunt due to missuture of atrial septal flap to the Eustachian valve which was mistaken as lower edge of the defect [7–9].

## Methods

**Fig. 1:**
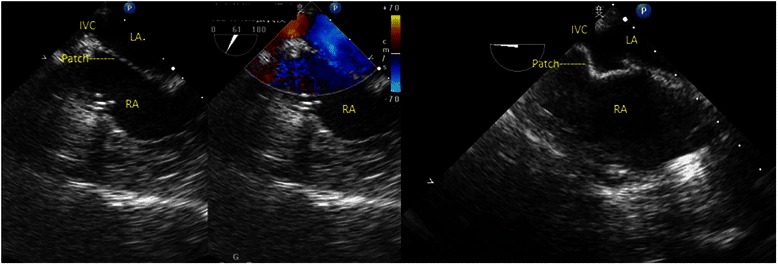
TEE showed an IVC-left atrium diversion soon after the repair. LA left atrium; RA right atrium; IVC inferior vena cava

We operated on one inferior SVD patient complicated with partial anomalous pulmonary venous return. A residual IVC - left atrial shunt were discovered by intraoperative transesophageal echocardiography soon after the surgery as the patient became cyanotic and hypoxic (Fig. 1). In order to improve access to the most inferior part of the defect, we reestablished the cardiopulmonary bypass through superior vena cava and right femoral vein cannulation, removed the originally missutured patch and repatched the defect.

In the management of this patients, we recognized that the main point of its surgical repair is the clear display of the inferior border of SVD and its surrounding structures. It is the IVC cannulation and the tightened tourniquet that hindered surgeons from getting a clear recognition of the surrounding structures. A more suitable and feasible cannulation technique to ensure exposure of the defect might be bicaval cannulation without snaring the IVC. We put it into practice in subsequent 12 patients with inferior SVD and proved successful.

## Results

**Fig. 2:**
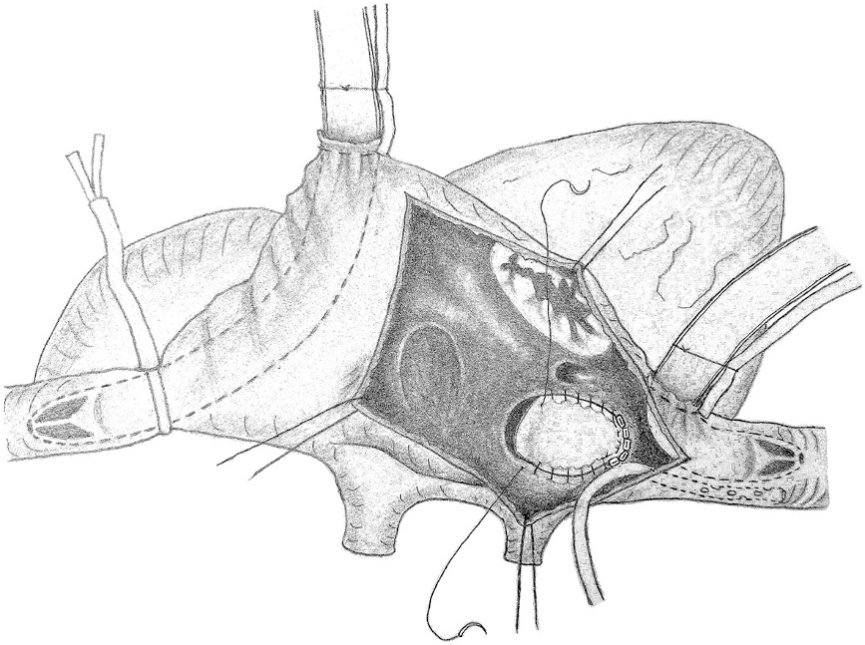
When conventional bicaval cannulation were applied, leaving the IVC unsnared can also facilitate to get a better exposure of the lower part of the SVD and IVC orifice. An intracardiac suction tube was placed into the IVC orifice to help blood drainage of the IVC

The essential skills of this technique are : bicaval cannulation was performed and SVC was routinely blocked with tightened tape, but the IVC leaving unsnared without any inferior tourniquet; right atriotomy was performed and an intracardiac suction tube was placed into the IVC orifice to obtain a clean surgical field in case that some blood would flow back into the right atrium during the CPB; after a clear inspection of the lower edge of atrial septal defect, IVC orifice, Eustachian valve, pulmonary vein insertion and surrounding structures, 4–6 stitches of 4–0 prolene pledgetted mattress sutures were used along the back side of the IVC orifice, then pushed the patch down and completed the repair with running sutures (Fig. 2).

## Discussion

There were reports about unsnared IVC techniques in particular situations with complex congenital anomaly or severe pericardial adhesions. In their depiction, femoral vein cannulation and unsnared IVC was applied to facilitate the surgical operations [10]. However, considering femoral vein cannulation need more operative procedures like additional preparation of groin area and dissection of femoral artery which may prolong the operating time, our experience indicated that the IVC could also be left unsnared while using conventional bicaval cannulation technique.

## Conclusions

In conclusion, for the surgical approach of inferior SVD patients, especially those not clearly diagnosed preoperatively, the conventional bicaval cannulation and the tightened IVC tourniquet may lead to shrinkage, folding and poor exposure of the lower part of SVD as well as the IVC opening. On the other hand, femoral vein cannulation need more operative procedures and is time consuming. In this situation, there is no need to remove the IVC cannulation and recannulate the femoral vein, just leaving the IVC unsnared can also facilitate to get a better exposure of the lower part of the SVD and IVC orifice, thus avoiding postoperative IVC - left atrial shunt and other surgical mistakes.

## Abbreviations

CPB: cardiopulmonary bypass
IVC: inferior vena cava
SVD: sinus venosus defect
SVC: superior vena cava
TEE: transesophageal echocardiography
TTE: transthoracic echocardiography
